# Supplementary material for: Comparative Evaluation of Diagnostic Tests for Brucellosis in Humans and Animals: A Meta-Analytical Approach
Source: Vet Sci. 2025 Jul 3;12(7):638. doi: 10.3390/vetsci12070638 (PMC12298791; doi:10.3390/vetsci12070638)
Supplement: Supplementary file 1 [file vetsci-12-00638-s001.zip › vetsci-3654408-supplementary.pdf]

Table S1: Comprehensive list of records included in the meta-analysis.

RefN	*Reference*	*Country*	*Specie*	*Sample*	*Tested*	*DxTest*	*Positive*	*Brucella species investigated*	*Quality score*	*Quality level*
1	Waringa et al., (2023) [43]	Kenya	Human	Serum	166	RBPT	17	B. abortus	6	High
						FBAT	44			
2	Luaces et al., (2023) [175]	Germany	Pig	Tissue	681	Culture	5	B. suis	5	Moderate
						PCR	8			
3	Elhaig et al., 2023 [87]	Egypt	Cattle	Serum	989	RBPT	205	B. abortus	8	High
						ELISA	234			
4	Abnaroodheleh et al., (2023) [88]	Iran	Cattle	Serum	935	RBPT	186	B. abortus, B. melitensis	6	High
						SAT	63			
						2-ME	48			
						ELISA	132			
5	Alhussain et al., (2022) [132]	Qatar	Camel	Serum	248	RBPT	52		7	High
						ELISA	55			
6	Kakooza S et al., 2022 [117]	Uganda	Sheep	Serum	66	RBPT	1		7	High
						ELISA	1			
			Goat		221	RBPT	17			
						ELISA	18			
7	Al-Afifi et al., (2022) [176]	Yemen	Cattle	Milk	262	MRT	8	B. abortus, B. melitensis	5	Moderate
						ELISA	9			
						Culture	1			
			Sheep		373	MRT	39			
						ELISA	37			

						Culture	8			
			Goat		288	MRT	19			
						ELISA	18			
						Culture	3			
8	Tialla et al., (2022) [118]	Burkina Faso	Sheep	Serum	300	RBPT	9		7	High
						ELISA	18			
			Goat		300	RBPT	3			
						ELISA	13			
9	Batrinou et al., (2022) [114]	Greece	Sheep	Blood	88	RBPT	10	B. abortus	5	Moderate
						PCR	0			
			Cattle		48	RBPT	2			
						PCR	2			
10	Almashhadany et al., 2022 [45]	Iraq	Human	Blood	325	RBPT	40	B. abortus, B. melitensis	7	High
						Culture	31			
11	Al-Marzooqi et al., 2022 [177]	Oman	Multi-species (ruminants)	Serum	434	RBPT	21		7	High
						CFT	13			
						I-ELISA	35			
12	Getahun et al., 2022 [47]	Ethiopia	Human	Serum	166	RBPT	7		6	High
						CFT	2			
13	Hussain et al., 2022 [89]	Pakistan	Cattle	Serum	570	RBPT	67	B. abortus	6	High
						ELISA	61			
						PCR	55			
14	Samkange et al., 2022 [116]	Namibia	Cattle	Serum	17	RBPT	0		5	Moderate
						CFT	0			

				Tissue	18	Culture	0			
						PCR	0			
			Goat	Serum	52	RBPT	12			
						CFT	12			
15	Obaidat et al., 2022 [48]	Jordan	Human	Serum	938	RBPT	56		8	High
						BRUCELLACAPT	9			
16	ZareBidaki et al., 2022 [178]	Iran	Multi-species (ruminants)	Serum	100	SAT	23	B. abortus, B. melitensis	7	High
						2-ME	23			
						PCR	37			
17	ŠERIĆ-HARAČIĆ et al., 2022 [179]	Bosnia and Herzegovina	Multi-species (ruminants)	Serum	2250	RBPT	303		6	High
						CFT	307			
						ELISA	183			
18	Islam et al., 2022 [49]	Pakistan	Goat	Serum	235	RBPT	32		7	High
						SPAT	38			
			Human		53	RBPT	2			
						SPAT	3			
19	Barreto et al., 2022 [180]	Brazil	Sheep	Serum	728	AGID	2	B. ovis	6	High
						ELISA	67			
20	Yousaf et al., 2021 [60]	Pakistan	Human	Serum	218	RBPT	37	B. abortus	7	High
						PCR	33			
21	Munsi et al., 2021 [119]	Bangladesh	Goat	Serum	190	RBPT	9		6	High
						c-ELISA	5			
22	Marouf et al., 2021 [181]	Iran	Sheep	Milk	214	PCR	37		6	High
						Culture	8			

			Goat		92	PCR	14			
						Culture	4			
			Multi-species (ruminants)	Cheese	266	PCR	61			
						Culture	12			
23	Niaz et al., 2021 [74]	Pakistan	Human	Serum	304	SAT	56		7	High
						ELISA	84			
24	Alam et al., 2021 [81]	Bangladesh	Human	Serum	400	LAT	28		5	Moderate
						PCR	22			
25	Zhou et al., 2021 [182]	China	Fox	Serum	736	RBPT	503	B. melitensis	5	Moderate
						SAT	308			
26	Tschopp et al., 2021 [83]	Ethiopia	Human	Serum	58	LFA	0		8	High
						ELISA	17			
27	Mubanga et al., 2021 [84]	Zambia	Human	Serum	153	I-ELISA	27		7	High
						c-ELISA	8			
28	Cadmus et al., 2021 [62]	Nigeria	Cattle	Serum	366	RBPT	8		7	High
						c-ELISA	19			
			Human		137	RBPT	1			
						c-ELISA	0			
29	Rahbarnia et al., 2021 [75]	Iran	Human	Blood	120	SAT	66		6	High
						Coombs	73			
						2-ME	43			
						Culture	10			
						PCR	55			
30	Govindasamy et al., 2021 [183]	South Africa	Human	Serum	230	RBPT	23		8	High

						BRUCELLACAPT	15			
						I-ELISA	48			
31	Ghugey et al., 2021 [63]	India	Human	Serum	382	RBPT	5		7	High
						I-ELISA	7			
32	Islam et al., 2021 [90]	Bangladesh	Cattle	Serum	158	RBPT	34		8	High
						c-ELISA	12			
33	Yusuf et al., 2021 [64]	Somalia	Human	Serum	307	RBPT	2		7	High
						c-ELISA	3			
34	Khan et al., 2021 [91]	Pakistan	Buffalo	Serum	112	RBPT	17		8	High
						I-ELISA	27			
			Cattle		108	RBPT	13			
						I-ELISA	20			
35	Chaudhari et al., 2021 [51]	India	Human	Serum	3051	RBPT	43		4	Moderate
						ELISA	56			
						SAT	12			
			Multi-species (ruminants)		9855	RBPT	898			
						ELISA	1152			
36	Galluzzo et al., 2021 [184]	Italy	Sheep	Serum	199	CFT	34	B. ovis	5	Moderate
						ELISA	42			
				Tissue	18	Histopathology	11			
						PCR	13			
37	Alumasa et al., 2021 [44]	Kenya	Human	Serum	180	RBPT	3		5	Moderate
						FBAT	24			
38	KATHIRIYA et al., 2021 [92]	India	Cattle	Serum	320	RBPT	58		6	High

						I-ELISA	60			
						LFA	42			
			Buffalo		182	RBPT	22			
						I-ELISA	24			
						LFA	16			
39	Saidu et al., 2021 [93]	India	Cattle	Serum	635	RBPT	321		8	High
						I-ELISA	308			
40	Yeni et al., 2021 [185]	Turkey	Multi-species (ruminants)	Serum	368	RBPT	42	B. melitensis	6	High
						SAT	37			
						CFT	36			
						PCR	36			
41	Abnaroodheleh et al., 2021 [120]	Iran	Sheep	Serum	101	RBPT	20	B. melitensis	6	High
						SAT	18			
						2-ME	15			
						I-ELISA	17			
			Goat		23	RBPT	16			
						SAT	10			
						2-ME	11			
						I-ELISA	12			
42	Awandkar et al., 2021 [121]	India	Goat	Serum	72	RBPT	25	B. melitensis	5	Moderate
						SAT	24			
						I-ELISA	24			
43	Suarez-Esquivel et al., 2021 [186]	Costa Rica	Dog	Serum	302	AGID	19	B. canis	6	High
						RBPT	5			
						c-ELISA	5			

				Mixed	187	AGID	149			
						Culture	29			
44	Akoko et al., 2021 [115]	Kenya	Cattle	Serum	228	RBPT	55	B. abortus, B. melitensis	8	High
						PCR	43			
45	Lukambagire et al., 2021 [65]	Tanzania	Human	Serum	218	RBPT	19		6	High
						c-ELISA	35			
46	Holt et al., 2021 [66]	India	Human	Serum	585	RBPT	17		8	High
						ELISA	56			
47	Munyua et al., 2021 [67]	Kenya	Human	Serum	236	RBPT	4		7	High
						I-ELISA	5			
						I-ELISA	39			
48	Nawaz et al., 2021 [187]	Pakistan	Buffalo	Milk	180	MRT	10		6	High
						I-ELISA	29			
			Goat		161	MRT	8			
						I-ELISA	3			
49	Camargo-Castañeda et al., 2021 [188]	Colombia	Dog	Tissue	8	PCR	4	B. canis	4	Moderate
						IHC	3			
50	Mol et al., 2020 [189]	Brazil	Dog	Serum	254	RBPT	19	B. canis	6	High
						CFT	39			
						AGID	16			
						PCR	31			
						MAT	25			
				Vaginal swab	52	Culture	6			
						PCR	6			
				Tissue	2	Culture	1			

						PCR	1			
51	Al-Sherida et al., 2020 [190]	Kuwait	Sheep	Tissue	16	Culture	10	B. melitensis	5	Moderate
						PCR	10			
				Milk	30	Culture	10			
						PCR	10			
52	Zhang et al., 2020 [94]	China	Cattle	Serum	1406	RBPT	95	B. melitensis	7	High
						c-ELISA	95			
						ELISA	95			
			Sheep		1590	RBPT	151			
						c-ELISA	151			
						ELISA	151			
53	Fero et al., 2020 [95]	Albania	Cattle	Serum	655	RBPT	147	B. abortus	5	Moderate
						ELISA	164			
						FPA	175			
54	Kalule et al., 2020 [82]	Uganda	Human	Serum	105	BAPA	25		5	Moderate
						Card test	25			
						SPAT	25			
						RIV	0			
			Dog		80	BAPA	1			
						Card test	1			
						SPAT	1			
						RIV	0			
55	Mangtani et al., 2020 [68]	India	Human	Serum	1801	RBPT	18		6	High
						I-ELISA	30			
56	Ukwueze et al., 2020 [96]	Nigeria	Cattle	Serum	221	RBPT	38		7	High
						ELISA	35			

			Goat		192	RBPT	29			
						ELISA	26			
			Sheep		60	RBPT	5			
						ELISA	14			
57	Dadar et al. 2020 [135]	Iran	Camel	Serum	2854	RBPT	10	B. melitensis	5	Moderate
						SAT	7			
						2-ME	6			
				Tissue	40	PCR	9			
						Culture	2			
58	Montagnaro et al., 2020 [191]	Italy	Pig	Serum	434	RBPT	22		7	High
						ELISA	58			
59	Leahy et al., 2020 [192]	India	Multi-species (ruminants)	Serum	432	RBPT	43		8	High
						ELISA	43			
60	Wainaina et al., 2020 [193]	Kenya	Cattle	Milk	50	ELISA	13	B. abortus, B. melitensis	6	High
						PCR	0			
61	Cadmus et al., 2020 [97]	Nigeria	Cattle	Serum	149	RBPT	17		7	High
						ELISA	10			
62	Naz et al., 2020 [98]	Pakistan	Buffalo	Serum	140	RBPT	0	B. abortus	6	High
						ELISA	3			
						PCR	29			
			Cattle		118	RBPT	0			
						ELISA	2			
						PCR	1			
63	Khan et al., 2020 [3]	Egypt	Camel	Serum	381	RBPT	59	B. abortus, B. melitensis, B. suis	7	High
						ELISA	87			

						ELISA	77			
						CFT	118			
64	Damke et al., 2020 [84]	India	Human	Serum	124	ELISA	12		6	High
						ELISA	28			
65	Bhandi et al., 2019 [128]	Zimbabwe	Goat	Serum	563	RBPT	0		8	High
						CFT	0			
66	Yang et al., 2019 [46]	China	Human	Serum	154	RBPT	92		6	High
						SAT	126			
						ELISA	147			
						Culture	5			
67	ElTahir et al., 2019 [99]	Oman	Goat	Serum	32	RBPT	0	B. melitensis	5	Moderate
						ELISA	1			
						CFT	7			
			Sheep		38	RBPT	1			
						ELISA	0			
						CFT	0			
			Cattle		1	RBPT	0			
						ELISA	0			
						CFT	0			
			Camel		17	RBPT	1			
						ELISA	1			
						CFT	1			
68	Dhason et al., 2019 [86]	India	Human	Serum	60	ELISA	9		6	High
						ELISA	1			
69	Falzon et al., 2019 [100]	Côte d'Ivoire	Cattle	Serum	44	RBPT	2		6	High

						FPA	4			
			Sheep		150	RBPT	4			
						FPA	16			
			Goat		38	RBPT	4			
						FPA	6			
70	Alamian et al., 2019 [194]	Iran	Camel	Milk	96	Culture	4	B. abortus	6	High
						PCR	14			
71	Nassar et al., (2019) [145]	Egypt	Buffalo	Serum	338	BPAT	8		6	High
						RBPT	7			
						CFT	5			
			Cattle		434	BPAT	12			
						RBPT	10			
						CFT	9			
72	Gioia et al., (2018) [101]	Ecuador	Cattle	Serum	410	RBPT	0		4	Moderate
						ELISA	0			
73	Harms et al., 2018 [195]	Canada	Bison	Serum	31	c-ELISA	0		5	Moderate
						Culture	0			
74	Scotter et al., 2018 [196]	Norway	Walrus	Serum	39	RBPT	9		5	Moderate
						I-ELISA	10			
75	Kanani et al., (2018) [122]	India	Sheep	Serum	1536	RBPT	195		6	High
						I-ELISA	230			
			Goat		2218	RBPT	318			
						I-ELISA	347			
76	Madut et al., (2018) [69]	Sudan	Cattle	Serum	893	RBPT	281		8	High
						c-ELISA	277			

			Human		87	RBPT	35			
						c-ELISA	29			
77	Awah-Ndukum et al., (2018) [70]	Cameroon	Human	Serum	816	RBPT	8		7	High
						I-ELISA	15			
			Cattle		590	RBPT	20			
						I-ELISA	35			
78	Sonekar et al., 2018 [129]	India	Sheep	Serum	157	RBPT	68	B. melitensis	5	Moderate
						ELISA	104			
79	ElTahir et al., (2018) [123]	Oman	Goat	Serum	324	RBPT	38	B. melitensis	7	High
						CFT	27			
						ELISA	62			
80	Ogugua et al., 2018 [102]	Nigeria	Cattle	Serum	513	RBPT	52		8	High
						ELISA	48			
				Milk	635	MRT	128			
						I-ELISA	113			
81	Adamu et al., 2018 [103]	Nigeria	Cattle	Serum	400	RBPT	74		7	High
						c-ELISA	27			
82	Kumar et al., (2018) [197]	India	Buffalo	Serum	920	RBPT	52		7	High
						LFA	53			
						SAT	50			
						ELISA	55			
83	Simten et al., (2018) [198]	Turkey	Sheep	Tissue	35	Culture	18	B. melitensis	5	Moderate
						IHC	21			
84	Hussein et al., 2018 [199]	Iraq	Sheep	Serum	30	ELISA	10	B. ovis	6	High

						PCR	14			
85	Proch et al., 2018 [52]	India	Human	Serum	279	RBPT	61		8	High
						SAT	67			
						ELISA	55			
						ELISA	150			
86	Shome et al., 2018 [130]	India	Sheep	Serum	300	RBPT	26	B. melitensis, B. ovis	4	Moderate
						ELISA	42			
						PCR	15			
87	Khan et al., (2018) [200]	Pakistan	Goat	Milk	100	MRT	6		7	High
						I-ELISA	76			
			Buffalo		100	MRT	11			
						I-ELISA	15			
			Multi-species (ruminants)		100	MRT	1			
						I-ELISA	42			
88	Hailat et al., (2018) [201]	Jordan	Sheep	Tissue	23	IHC	3	B. melitensis	4	Moderate
						PCR	5			
			Goat		2	IHC	0			
						PCR	0			
89	Keramat et al., 2017 [76]	Iran	Human	Serum	157	SAT	3		6	High
						Coombs test	5			
						2-ME	0			
						Culture	0			
90	Barkallah et al., 2017 [104]	Tunisia	Cattle	Serum	214	RBPT	52	B. abortus, B. melitensis	8	High
						ELISA	43			
			Sheep		164	RBPT	24			

						ELISA	14			
91	Aworh et al., (2017) [105]	Nigeria	Cattle	Serum	376	RBPT	21		7	High
						c-ELISA	2			
			Sheep		203	RBPT	18			
						c-ELISA	4			
			Goat		260	RBPT	51			
						c-ELISA	10			
92	Anjaneyareddy et al., 2017 [106]	India	Cattle	Serum	153	RBPT	12		6	High
						LFA	11			
			Buffalo		55	RBPT	2			
						LFA	2			
			Sheep		140	RBPT	18			
						LFA	15			
			Goat		219	RBPT	6			
						LFA	7			
			Pig		225	RBPT	88			
						LFA	74			
93	Eckstein et al., 2017 [202]	Brazil	Sheep	Serum	480	AGID	30	B. ovis	6	High
						ELISA	140			
				Urine	83	Culture	0			
						PCR	0			
				Semen	72	Culture	1			
						PCR	0			
94	Rahman et al., 2017 [203]	Bangladesh	Cattle	Tissue	5	Staining	0	B. abortus	5	Moderate
						Culture	0			
						PCR	0			

			Goat		10	Staining	0			
						Culture	0			
						PCR	0			
			Sheep		8	Staining	0			
						Culture	0			
						PCR	0			
			Cattle	Vaginal swab	4	Staining	0			
						Culture	0			
						PCR	0			
			Goat		10	Staining	0			
						Culture	0			
						PCR	0			
			Sheep		3	Staining	0			
						Culture	0			
						PCR	0			
			Cattle	Semen	5	Culture	0			
						PCR	1			
95	Baloch et al., 2017 [133]	Pakistan	Camel	Serum	100	RBPT	21		4	Moderate
						SAT	21			
						c-ELISA	13			
96	Khan et al., (2017) [77]	Pakistan	Human	Serum	200	SPAT	12	B. abortus, B. melitensis	6	High
						PCR	8			
			Cattle		200	SPAT	30			
						PCR	26			
97	Alves et al., 2017 [204]	Brazil	Sheep	Serum	119	BAPA	0	B. ovis	7	High
						AGID	7			
98	Altun et al., (2017) [205]	Turkey	Multi-species (ruminants)	Cheese	80	I-ELISA	13		4	Moderate

						PCR	18			
			Cattle	Milk	48	I-ELISA	8			
						PCR	9			
			Goat		65	I-ELISA	4			
						PCR	5			
			Sheep		65	I-ELISA	4			
						PCR	4			
99	Azam et al., 2017 [206]	Pakistan	Human	Serum	200	RBPT	89		5	Moderate
						PCR	41			
100	Sharma et al., (2016) [53]	India	Human	Serum	121	RBPT	12		6	High
						SAT	11			
						I-ELISA	20			
101	Germeraad et al., (2016) [54]	Gambia	Human	Serum	599	RBPT	1		6	High
						MAT	0			
						ELISA	0			
			Sheep		379	RBPT	14			
						ELISA	0			
			Goat		744	RBPT	0			
						ELISA	0			
102	Costa et al., (2016) [124]	Brazil	Sheep	Serum	124	RBPT	0	B. ovis	4	Moderate
						AGID	3			
						ELISA	29			
				Urine	124	Culture	0			
						PCR	4			
			Goat	Serum	34	RBPT	0			
						AGID	3			

						ELISA	11			
				Urine	32	Culture	0			
						PCR	0			
103	Pathak et al., 2016 [107]	India	Cattle	Serum	296	RBPT	90	B. abortus	6	High
						ELISA	123			
104	Purwar et al., (2016) [78]	India	Human	Serum	400	SAT	35		6	High
						2-ME	34			
						PCR	32			
						Culture	20			
105	Mangalgi et al., (2016) [55]	India	Human	Serum	2337	RBPT	223		5	Moderate
						SAT	106			
						2-ME	87			
106	Shirima et al., (2016) [71]	Tanzania	Human	Serum	82	RBPT	0		7	High
						ELISA	0			
107	Salih et al., 2016 [134]	Saudi Arabia	Camel	Serum	750	RBPT	49		8	High
						c-ELISA	24			
108	Praud et al., 2016 [108]	France	Cattle	Serum	4430	RBPT	5		4	Moderate
						CFT	1			
						SAT	1			
						I-ELISA	8			
						c-ELISA	76			
109	Cardona et al., 2016 [125]	El Salvador	Sheep	Serum	396	RBPT	4		4	Moderate
						I-ELISA	0			
			Goat		335	RBPT	0			
						I-ELISA	0			

110	Gwida et al., (2016) [109]	Egypt	Cattle	Serum	257	RBPT	24		5	Moderate
						ELISA	29			
						FPA	22			
						PCR	120			
111	Mahmood et al., (2016) [61]	Pakistan	Human	Serum	110	RBPT	5		6	High
						c-ELISA	10			
						FPA	34			
						PCR	42			
112	Raza et al., 2016 [207]	Pakistan	Cattle	Serum	300	RBPT	38		6	High
						ELISA	33			
113	Sadhu et al., (2015) [126]	India	Goat	Serum	515	RBPT	42		6	High
						SAT	41			
						I-ELISA	31			
			Sheep	Serum	485	RBPT	71			
						SAT	70			
						I-ELISA	57			
114	Tanner et al., 2015 [208]	Mozambique	Buffalo	Serum	62	RBPT	11		6	High
						ELISA	17			
115	Wareth et al., (2015) [110]	Egypt	Cattle	Serum	10	RBPT	10	B. abortus, B. melitensis	4	Moderate
						CFT	10			
						ELISA	10			
						PCR	10			
			Buffalo		5	RBPT	1			
						CFT	1			
						ELISA	1			

						PCR	5			
			Goat		9	RBPT	3			
						CFT	3			
						ELISA	3			
						PCR	9			
			Sheep		1	RBPT	1			
						CFT	1			
						ELISA	1			
						PCR	1			
116	Mangalgi et al., (2015) [56]	India	Human	Serum	1733	RBPT	182		7	High
						SAT	127			
						2-ME	102			
117	Fiasconaro et al., 2015 [131]	Italy	Sheep	Serum	1515	RBPT	1430		6	High
						CFT	1384			
						c-ELISA	1513			
						FPA	1454			
118	Mathew et al., (2015) [111]	Tanzania	Cattle	Serum	200	RBPT	43	B. abortus	4	Moderate
						I-ELISA	96			
				Milk	10	MRT	10			
						Culture	0			
			Goat	Serum	50	RBPT	0			
						I-ELISA	1			
			Sheep	Serum	35	RBPT	0			
						I-ELISA	2			
119	Barlozzari et al., (2015) [209]	Italy	Pig	Serum	28	RBPT	25	B. suis	4	Moderate
						CFT	22			

120	Fatima et al., 2015 [136]	Pakistan	Camel	Serum	200	RBPT	10		7	High
						c-ELISA	4			
121	Shah et al., 2015 [210]	Pakistan	Sheep	Serum	300	RBPT	14		6	High
						I-ELISA	13			
122	Abakar et al., 2014 [112]	Chad	Cattle	Serum	561	RBPT	32		8	High
						ELISA	67			
123	Wu et al., 2014 [211]	United States of America	Dolphin	Tissue	37	Culture	9	B. ceti, B. pinnipedialis	6	High
						PCR	27			
124	Wareth et al., (2014) [212]	Egypt	Cattle	Milk	72	I-ELISA	18	B. abortus, B. melitensis	5	Moderate
						PCR	9			
			Buffalo		128	I-ELISA	13			
						PCR	7			
125	Martino et al., 2014 [213]	Argentina	Rodent	Serum	176	ELISA	0		6	High
						CFT	0			
126	Asif et al., 2014 [79]	Pakistan	Human	Serum	95	SAT	37		6	High
						PCR	14			
127	YÜKSEKKAYA et al., 2013 [57]	Turkey	Human	Serum	1000	RBPT	0	B. canis	6	High
						SAT	34			
						MPAT	22			
128	Dean et al., 2013 [72]	Togo	Human	Serum	683	RBPT	3	B. abortus	8	High
						ELISA	5			
			Cattle		1060	RBPT	55			
						ELISA	90			
129	Rahman et al., 2013 [127]	Bangladesh	Goat	Serum	636	RBPT	5		6	High

						SAT	5			
						ELISA	32			
			Sheep		1044	RBPT	26			
						SAT	23			
						ELISA	16			
130	Peeridogaheh et al., 2013 [80]	Iran	Human	Serum	89	Brucellacapt	49		5	Moderate
						ELISA	23			
						ELISA	43			
131	Aworh et al., 2013 [73]	Nigeria	Human	Serum	224	RBPT	38		8	High
						ELISA	40			
132	Priyadarshini et al., 2013 [58]	India	Cattle	Serum	258	RBPT	11		6	High
						SAT	6			
						I-ELISA	21			
			Human		88	RBPT	5			
						SAT	3			
133	Gul et al., 2013 [214]	Pakistan	Horse	Serum	308	RBPT	62		6	High
						SAT	50			
134	Szulowski et al., 2013 [113]	Poland	Cattle	Serum	176	RBPT	176	B. suis	5	Moderate
						SAT	176			
						CFT	170			
135	Din et al., 2013 [59]	Pakistan	Human	Serum	150	RBPT	14		6	High
						SAT	9			
						SPAT	11			
			Goat		150	RBPT	20			
						SAT	14			
						SPAT	17			

Diagnosis of Brucellosis

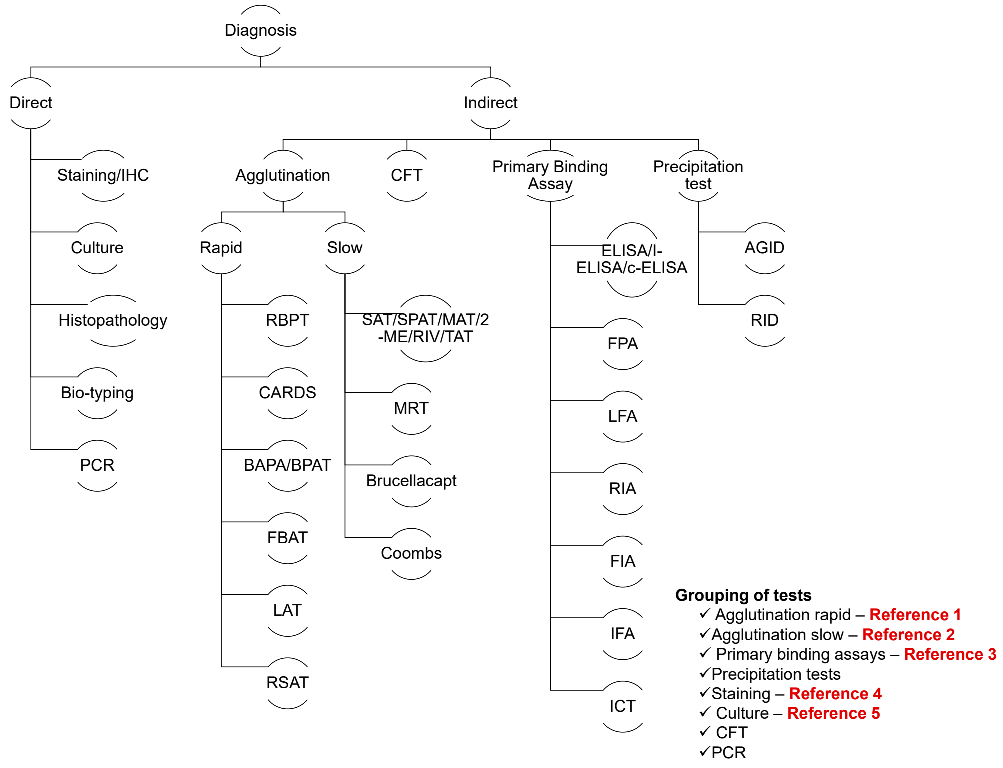

Figure S1. The tests used for diagnosing brucellosis in humans and animals are categorized into two primary groups as described by [33,42]. The tests are further divided into different subgroups with little modifications. The test grouping along with reference number at the bottom right corner indicates the sequence of test used as reference for the test comparison meta-analysis.

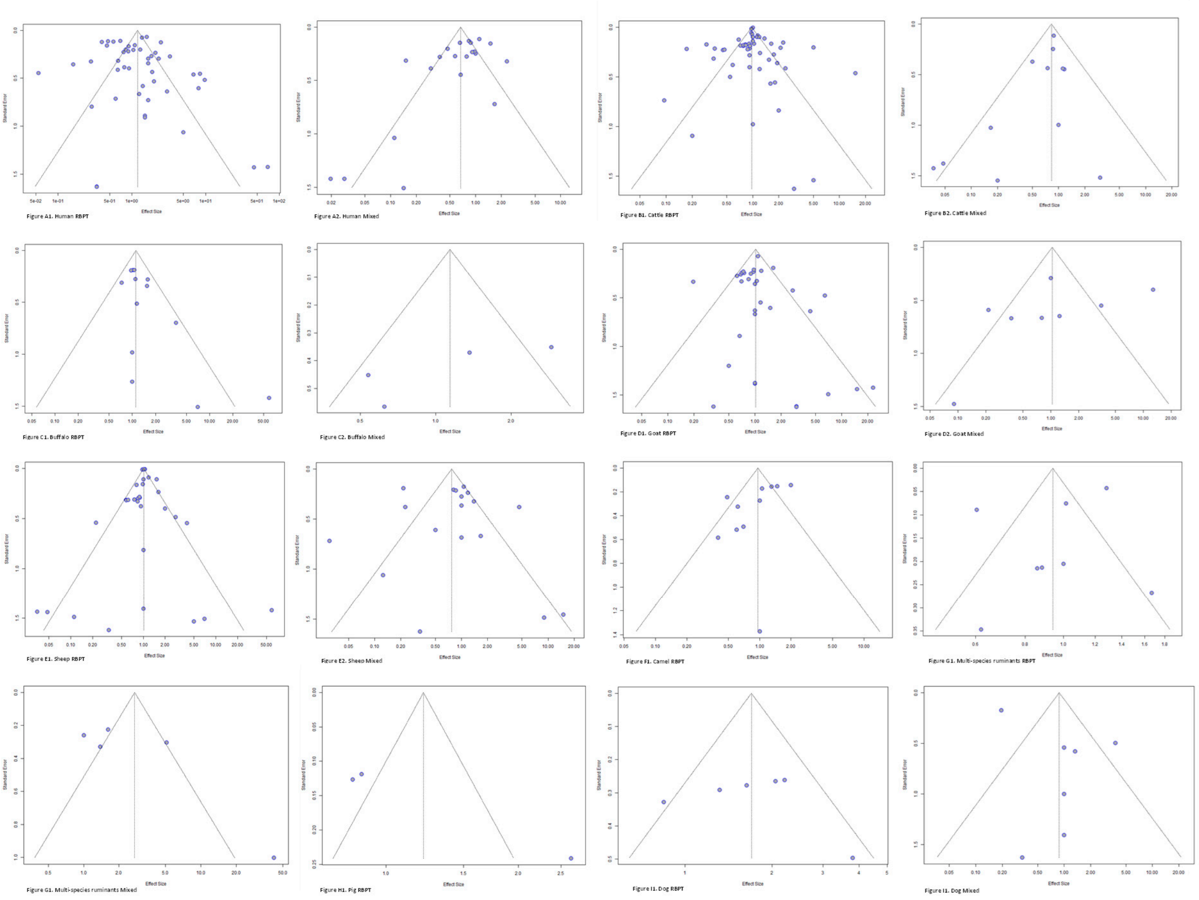

Figure S2. Species-wise funnel plots illustrating the publication bias among studies included in the meta-analysis. The “RBPT-type” plots represent comparisons where the Rose Bengal Plate Test (RBPT) was used as the control test, while the “mixed-type” plots correspond to comparisons where other diagnostic tests served as controls in the absence of RBPT, following the prioritization hierarchy outlined in the materials and methods section.

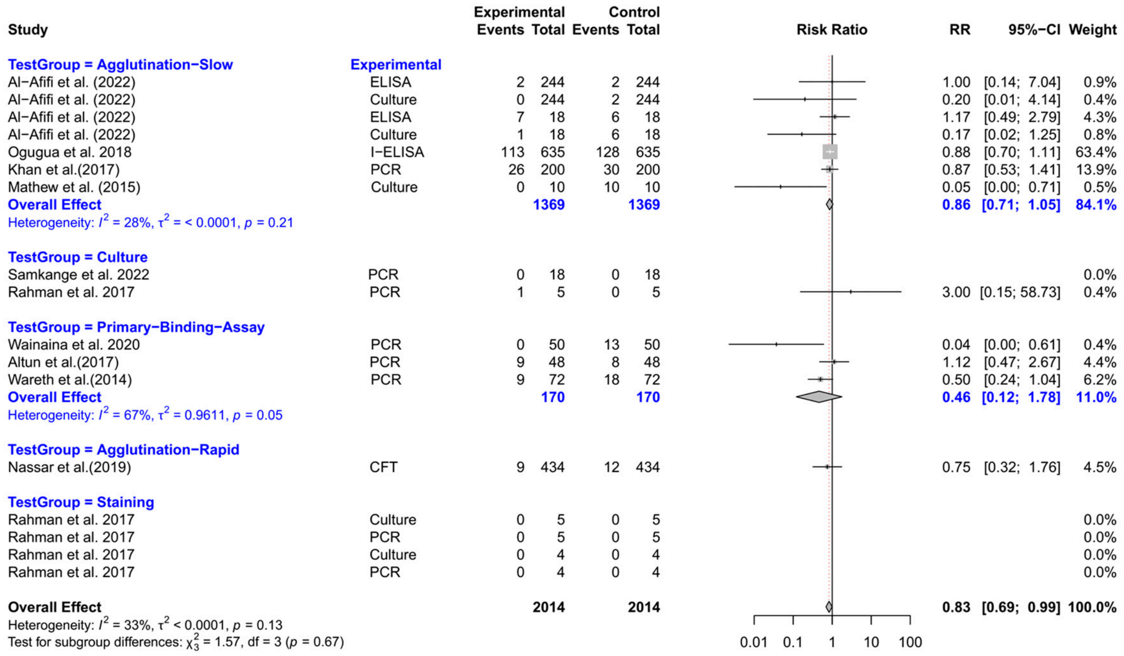

Figure S3. The forest plot represents the relative risk (RR) of various tests [experimental] compared with five different test groups [control] for diagnosing brucellosis in cattle. A total of 17 comparisons are depicted in the forest plot. Five comparisons were excluded because they had zero positive events in both groups i.e. experiment and control. The subgroup analyses revealed that the RRs for diagnosing brucellosis did not differ significantly between experimental tests and control groups. [77,102,111,116,176,193, 203,205,212]

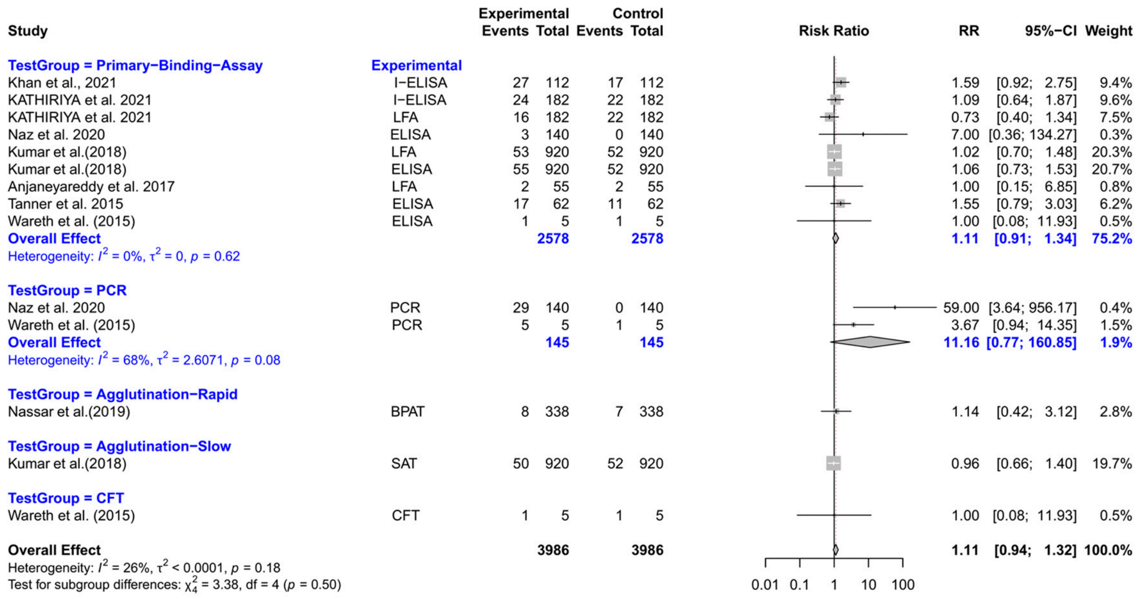

Figure S4. The forest plot represents the relative risk (RR) of 5 different test groups [experimental] compared with the Rose Bengal plate test (RBPT) [control] for diagnosing brucellosis in buffaloes. A total of 14 comparisons are depicted in the forest plot. Overall, the RRs for diagnosing brucellosis when compared to RBPT did not differ significantly [RR (95% CI) = 1.11 (0.94-1.32), I^2 = 26 %]. The subgroup analyses revealed that the primary binding assays, PCR, rapid agglutination tests, slow agglutination tests, and CFT did not differ significantly from RBPT. [91,92,98,106,110,197,208]

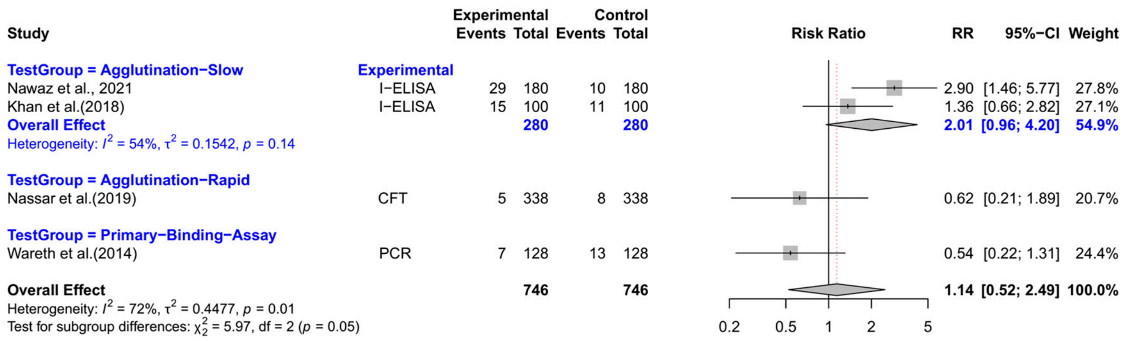

Figure S5. The forest plot represents the relative risk (RR) of various tests [experimental] compared to three different test groups [control] for diagnosing brucellosis in buffaloes. A total of 4 comparisons are depicted in the forest plot. The subgroup analyses revealed that the RRs for brucellosis diagnosis did not differ significantly when experimental tests were compared with control groups. [187,200,212]

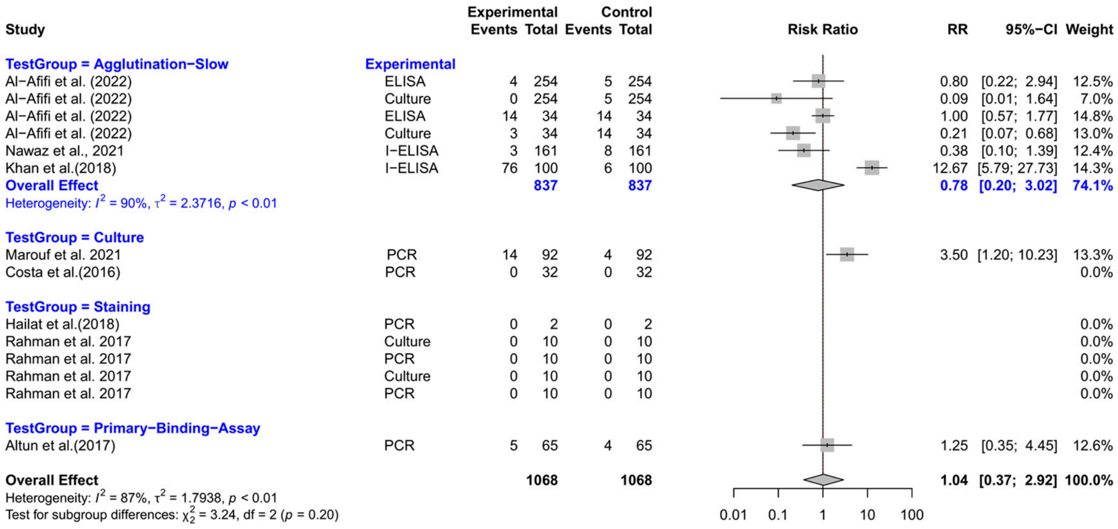

Figure S6. The forest plot represents the relative risk (RR) of various tests [experimental] compared to four different test groups [control] for diagnosing brucellosis in goats. A total of 14 comparisons are depicted in the forest plot. Six comparisons were excluded because they had zero positive events in both groups, i.e., experiment and control. The subgroup analyses revealed that the RRs for brucellosis diagnosis did not differ significantly when experimental tests were compared with control groups. [124,176,181,187,200,201,203,205]

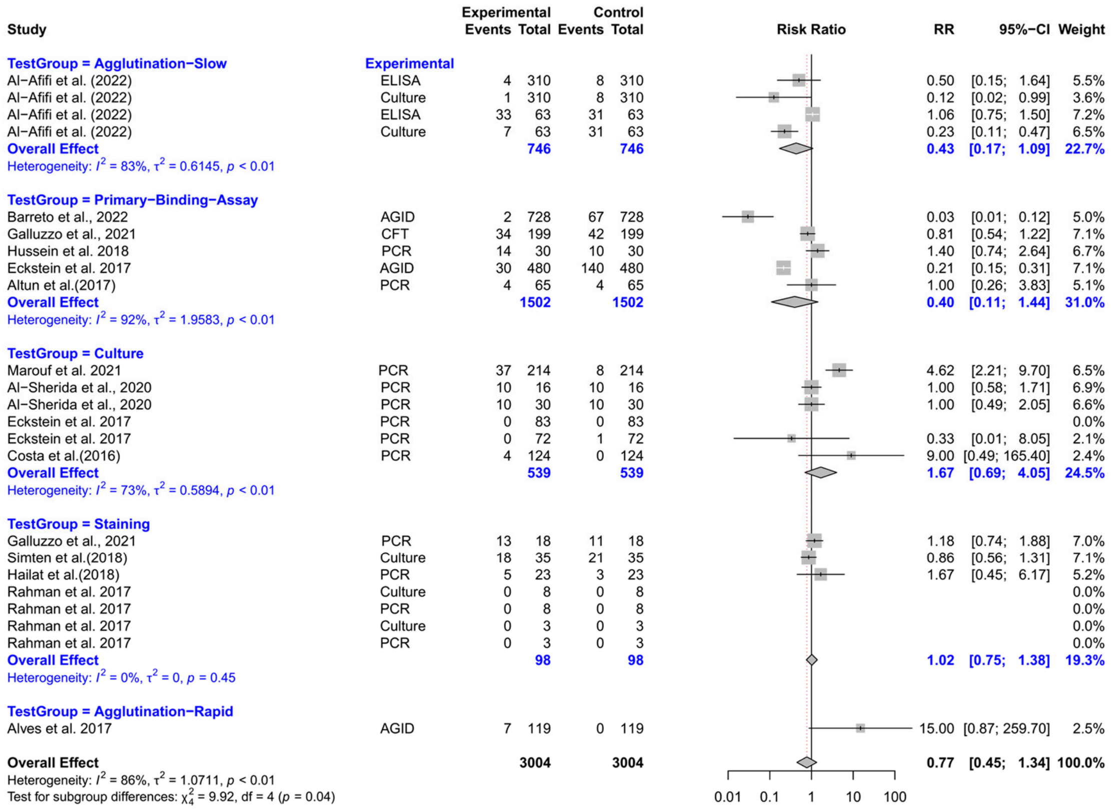

Figure S7. The forest plot represents the relative risk (RR) of various tests [experimental] compared with five different test groups [control] for diagnosing brucellosis in sheep. A total of 23 comparisons are depicted in the forest plot. Five comparisons were excluded because they had zero positive events in both groups, i.e., experiment and control. The subgroup analyses revealed that the RRs for diagnosing brucellosis did not differ significantly when experimental tests were compared with control groups. [124,176,180,181,184,190,198,199,201,202,203,204,205]

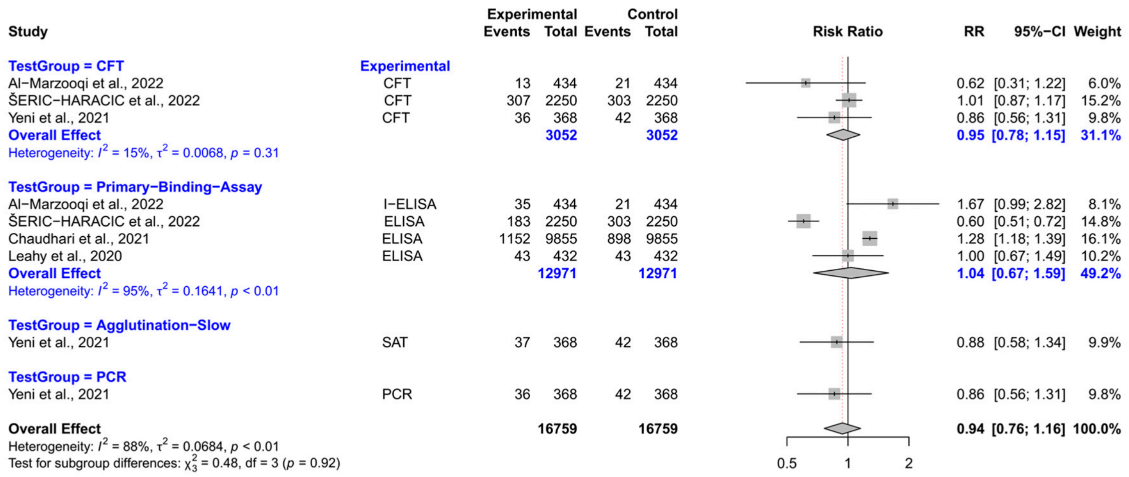

Figure S8. The forest plot represents the relative risk (RR) of four different test groups [experimental] compared with the Rose Bengal plate test RBPT [control] for diagnosing brucellosis in samples of multi-species (ruminants). A total of 9 comparisons are depicted in the forest plot. Overall, the RRs for diagnosing brucellosis when compared to RBPT did not differ significantly [RR (95% CI) = 0.94 (0.76-1.16), I^2 = 88 %]. The subgroup analyses revealed that the CFT, primary binding assays, slow agglutination tests, and PCR did not differ significantly from RBPT. [51,177,179,185,192]

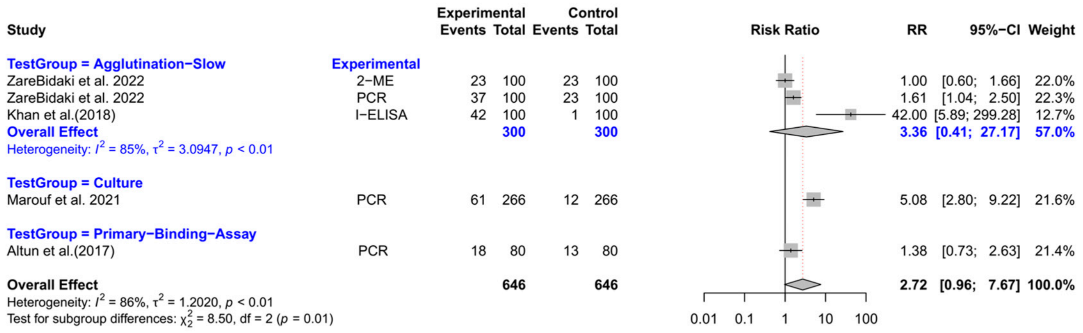

Figure S9. The forest plot represents the relative risk (RR) of various tests [experimental] compared with three different test groups [control] for diagnosing brucellosis in samples of multi-species (ruminants). A total of five comparisons are depicted in the forest plot. The subgroup analyses revealed that the RRs for diagnosing brucellosis did not differ significantly when experimental tests were compared with control groups. [178,181,200,205]

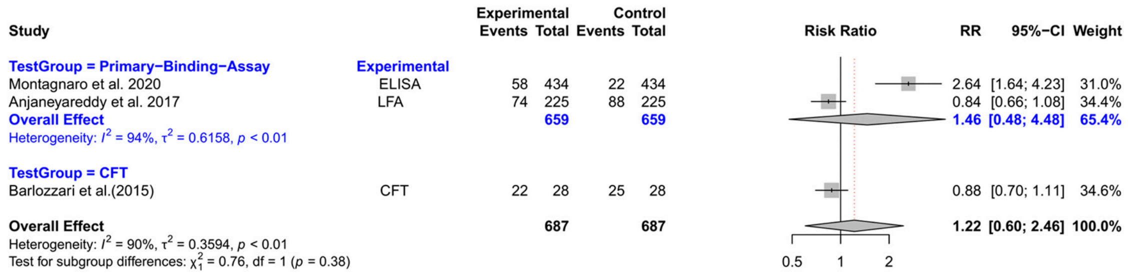

Figure S10. The forest plot represents the relative risk (RR) of two different test groups [experimental] compared with the Rose Bengal plate test (RBPT) [control] for diagnosing brucellosis in pigs. A total of three comparisons are depicted in the forest plot. Overall, the RRs for diagnosing brucellosis when compared to RBPT did not differ significantly [RR (95% CI) = 1.22 (0.60-2.46), I^2 = 90 %]. The subgroup analyses revealed that the primary binding assays and CFT did not differ significantly from RBPT. [106,191,209]

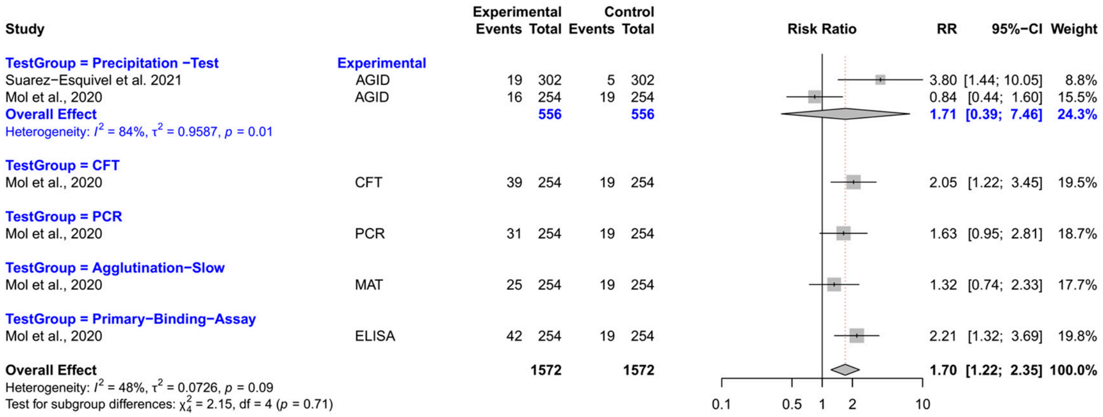

Figure S11. The forest plot represents the relative risk (RR) of five different test groups [experimental] compared with the Rose Bengal plate test (RBPT) [control] for diagnosing brucellosis in dogs. A total of six comparisons are depicted in the forest plot. Overall, the RR for diagnosing brucellosis was significantly higher compared to RBPT [RR (95% CI) = 1.70 (1.22-2.35), I^2 = 48 %]. However, the subgroup analyses revealed that the precipitation tests, CFT, PCR, slow agglutination tests, and primary binding assays did not differ significantly from RBPT. [186,189]

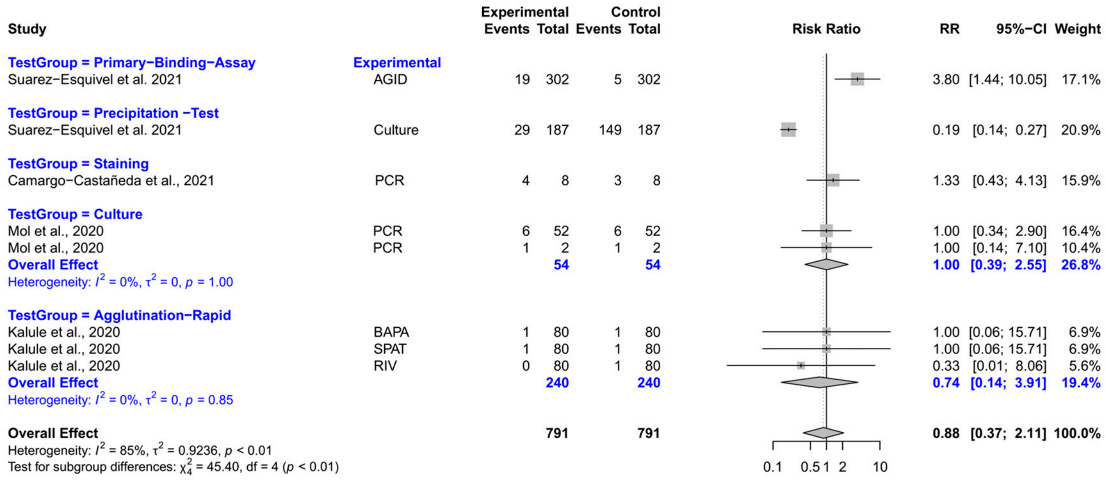

Figure S12. The forest plot represents the relative risk (RR) of various diagnostic tests [experimental] compared with five different diagnostic test groups [control] for diagnosing brucellosis in dogs. A total of eight comparisons are depicted in the forest plot. The subgroup analyses revealed that the RRs for diagnosing brucellosis did not differ significantly when experimental tests were compared with control groups. [82,186,188,189]

Study	Experimental Events	Experimental Total	Control Events	Control Total	RR	95%−CI	Weight
**QualityLevel = High**							
Waringa et al.,(2023)	9	52	3	52	3.00	[0.86; 10.46]	1.8%
Waringa et al.,(2023)	30	84	13	84	2.31	[1.30; 4.11]	2.4%
Waringa et al.,(2023)	5	30	1	30	5.00	[0.62; 40.28]	1.2%
Almashhadany et al., 2022	31	325	40	325	0.78	[0.50; 1.21]	2.5%
Getahun et al., 2022	2	166	7	166	0.29	[0.06; 1.36]	1.6%
Obaidat et al. 2022	9	938	56	938	0.16	[0.08; 0.32]	2.3%
Islam et al., 2022	3	53	2	53	1.50	[0.26; 8.62]	1.4%
Yousaf et al., 2021	33	218	37	218	0.89	[0.58; 1.37]	2.5%
Cadmus et al., 2021	0	137	1	137	0.33	[0.01; 8.11]	0.7%
Govindasamy et al. 2021	15	230	23	230	0.65	[0.35; 1.22]	2.4%
Govindasamy et al. 2021	48	230	23	230	2.09	[1.31; 3.31]	2.5%
Ghugey et al., 2021	7	382	5	382	1.40	[0.45; 4.37]	1.9%
Yusuf et al., 2021	3	307	2	307	1.50	[0.25; 8.91]	1.4%
Lukambagire et al., 2021	35	218	19	218	1.84	[1.09; 3.12]	2.5%
Holt et al., 2021	56	585	17	585	3.29	[1.94; 5.60]	2.5%
Munyua et al., 2021	5	236	4	236	1.25	[0.34; 4.60]	1.8%
Munyua et al., 2021	39	236	4	236	9.75	[3.54; 26.85]	2.0%
Mangtany et al., 2020	30	1801	18	1801	1.67	[0.93; 2.98]	2.4%
Yang et al. 2019	126	154	92	154	1.37	[1.18; 1.59]	2.6%
Yang et al. 2019	147	154	92	154	1.60	[1.40; 1.83]	2.6%
Yang et al. 2019	5	154	92	154	0.05	[0.02; 0.13]	2.2%
Madut et al.(2018)	29	87	35	87	0.83	[0.56; 1.23]	2.5%
Awah−Ndukum et al.(2018)	15	816	8	816	1.88	[0.80; 4.40]	2.2%
Proch et al. 2018	67	279	61	279	1.10	[0.81; 1.49]	2.6%
Proch et al. 2018	55	279	61	279	0.90	[0.65; 1.25]	2.6%
Proch et al. 2018	150	279	61	279	2.46	[1.92; 3.15]	2.6%
Sharma et al.(2016)	11	121	12	121	0.92	[0.42; 2.00]	2.3%
Sharma et al.(2016)	20	121	12	121	1.67	[0.85; 3.26]	2.4%
Germeraad et al.(2016)	0	599	1	599	0.33	[0.01; 8.17]	0.7%
Germeraad et al.(2016)	0	599	1	599	0.33	[0.01; 8.17]	0.7%
Shirima et al.(2016)	0	82	0	82			0.0%
Mahmood et al.(2016)	10	110	5	110	2.00	[0.71; 5.66]	2.0%
Mahmood et al.(2016)	34	110	5	110	6.80	[2.76; 16.74]	2.2%
Mahmood et al.(2016)	42	110	5	110	8.40	[3.45; 20.43]	2.2%
Mangalgi et al.(2015)	127	1733	182	1733	0.70	[0.56; 0.87]	2.6%
Mangalgi et al.(2015)	102	1733	182	1733	0.56	[0.44; 0.71]	2.6%
YÜKSEKKAYA et al. 2014	34	1000	0	1000	69.00	[4.24; 1123.89]	0.8%
YÜKSEKKAYA et al. 2014	22	1000	0	1000	45.00	[2.73; 740.80]	0.8%
Dean et al. 2013	5	683	3	683	1.67	[0.40; 6.95]	1.7%
Aworh et al. 2013	40	224	38	224	1.05	[0.70; 1.58]	2.5%
Priyadarshini et al. 2013	3	88	5	88	0.60	[0.15; 2.43]	1.7%
Din et al. 2013	9	150	14	150	0.64	[0.29; 1.44]	2.2%
Din et al. 2013	11	150	14	150	0.79	[0.37; 1.67]	2.3%
**Overall Effect**		**17043**		**17043**	**1.30**	**[0.95; 1.78]**	**85.4%**
Heterogeneity: I^2 = 87%, τ^2 = 0.8159, p < 0.01							
**QualityLevel = Moderate**							
Chaudhari et al., 2021	56	3051	43	3051	1.30	[0.88; 1.93]	2.5%
Chaudhari et al., 2021	12	3051	43	3051	0.28	[0.15; 0.53]	2.4%
Alumasa et al., 2021	24	180	3	180	8.00	[2.45; 26.10]	1.9%
Azam et al. 2017	41	200	89	200	0.46	[0.34; 0.63]	2.6%
Mangalgi et al.(2016)	106	2337	223	2337	0.48	[0.38; 0.59]	2.6%
Mangalgi et al.(2016)	87	2337	223	2337	0.39	[0.31; 0.50]	2.6%
**Overall Effect**		**11156**		**11156**	**0.73**	**[0.31; 1.73]**	**14.6%**
Heterogeneity: I^2 = 90%, τ^2 = 1.0918, p < 0.01							
**Overall Effect**		**28199**		**28199**	**1.19**	**[0.88; 1.61]**	**100.0%**
Heterogeneity: I^2 = 91%, τ^2 = 0.8867, p < 0.01							
Test for subgroup differences: χ^2_1 = 1.52, df = 1 (p = 0.22)							

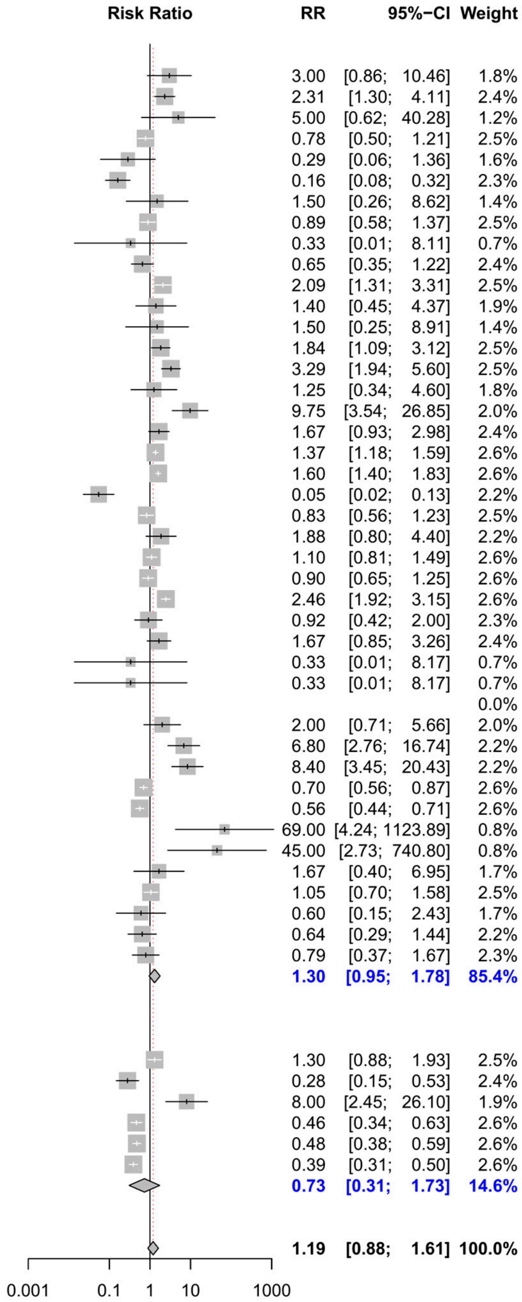

Figure S13. The forest plot represents the subgroup meta-analysis by study quality, with RBPT as reference (control), in humans. [1,10,12,15,18,20,28,30,31,33,35,37,45-47,55,66,75,76,84,99,100,105,110,115,126,127,130,131,134]

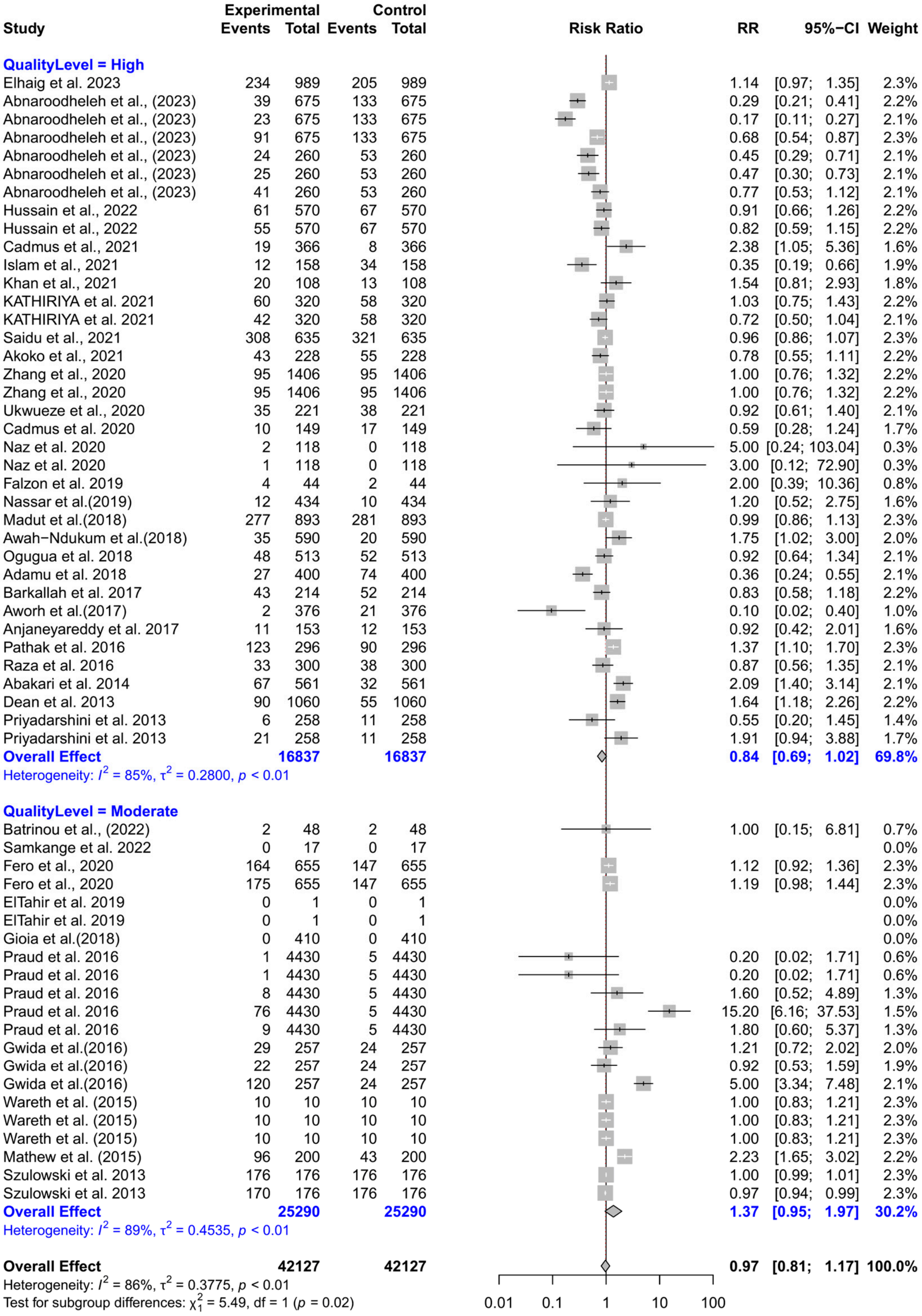

Figure S14. The forest plot represents the subgroup meta-analysis by study quality, with RBPT as reference (control), in cattle. [3,4,9,13,14,28,32,34,38,39,44,52,53,56,61,62,67,69,71,75,76,79,80,89-91,102,107,109,114,117,121,127,131,133]

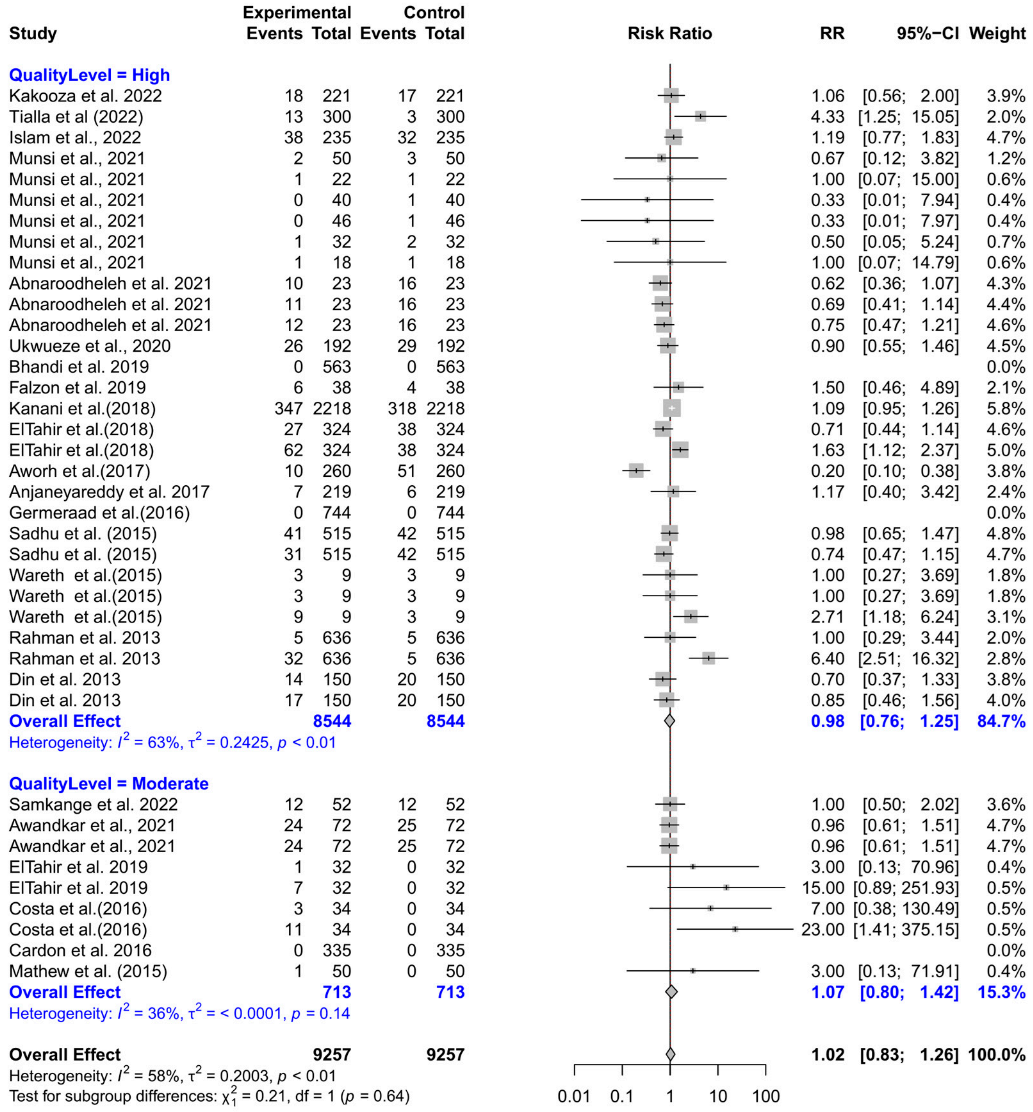

Figure S15. The forest plot represents the subgroup meta-analysis by study quality, with RBPT as reference (control), in goats.

[6,8,14,18,21,41,42,56,65,67,69,74,78,90,91,100,101,108,112,114,117,128,134]

Study	Experimental Events	Experimental Total	Control Events	Control Total	RR	95%−CI	Weight
**QualityLevel = High**							
Kakooza et al. 2022	1	66	1	66	1.00	[0.06; 15.65]	0.1%
Tialla et al (2022)	18	300	9	300	2.00	[0.91; 4.38]	1.4%
Abnaroodheleh et al. 2021	18	101	20	101	0.90	[0.51; 1.60]	2.4%
Abnaroodheleh et al. 2021	15	101	20	101	0.75	[0.41; 1.38]	2.2%
Abnaroodheleh et al. 2021	17	101	20	101	0.85	[0.47; 1.53]	2.3%
Zhang et al., 2020	151	1590	151	1590	1.00	[0.81; 1.24]	7.1%
Zhang et al., 2020	151	1590	151	1590	1.00	[0.81; 1.24]	7.1%
Ukwueze et al., 2020	14	60	5	60	2.80	[1.08; 7.29]	1.0%
Falzon et al. 2019	16	150	4	150	4.00	[1.37; 11.69]	0.8%
Kanani et al.(2018)	230	1536	195	1536	1.18	[0.99; 1.41]	7.9%
Barkallah et al. 2017	14	164	24	164	0.58	[0.31; 1.09]	2.1%
Aworh et al.(2017)	4	203	18	203	0.22	[0.08; 0.65]	0.8%
Anjaneyareddy et al. 2017	15	140	18	140	0.83	[0.44; 1.59]	2.0%
Germeraad et al.(2016)	0	379	14	379	0.03	[0.00; 0.58]	0.1%
Sadhu et al.(2015)	70	485	71	485	0.99	[0.73; 1.34]	5.4%
Sadhu et al.(2015)	57	485	71	485	0.80	[0.58; 1.11]	5.0%
Fiasconaro et al., 2015	1384	1515	1430	1515	0.97	[0.95; 0.99]	10.4%
Fiasconaro et al., 2015	1513	1515	1430	1515	1.06	[1.04; 1.07]	10.4%
Fiasconaro et al., 2015	1454	1515	1430	1515	1.02	[1.00; 1.03]	10.4%
Shah et al. 2015	13	300	14	300	0.93	[0.44; 1.94]	1.6%
Rahman et al. 2013	23	1044	26	1044	0.88	[0.51; 1.54]	2.5%
Rahman et al. 2013	16	1044	26	1044	0.62	[0.33; 1.14]	2.2%
**Overall Effect**		**14384**		**14384**	**1.01**	**[0.96; 1.05]**	**85.4%**
Heterogeneity: $I^2 = 79\%$, $\tau^2 = 0.0019$, $p < 0.01$							
**QualityLevel = Moderate**							
Batrinou et al., (2022)	0	88	10	88	0.05	[0.00; 0.80]	0.1%
ElTahir et al. 2019	0	38	1	38	0.33	[0.01; 7.93]	0.1%
ElTahir et al. 2019	0	38	1	38	0.33	[0.01; 7.93]	0.1%
Sonekar et al 2018	104	157	68	157	1.53	[1.24; 1.89]	7.2%
Shome et al. 2018	42	300	26	300	1.62	[1.02; 2.56]	3.3%
Shome et al. 2018	15	300	26	300	0.58	[0.31; 1.07]	2.2%
Costa et al.(2016)	3	124	0	124	7.00	[0.37; 134.11]	0.1%
Costa et al.(2016)	29	124	0	124	59.00	[3.65; 954.97]	0.1%
Cardona et al. 2016	0	396	4	396	0.11	[0.01; 2.06]	0.1%
Wareth et al. (2015)	1	1	1	1	1.00	[0.20; 4.95]	0.4%
Wareth et al. (2015)	1	1	1	1	1.00	[0.20; 4.95]	0.4%
Wareth et al. (2015)	1	1	1	1	1.00	[0.20; 4.95]	0.4%
Mathew et al. (2015)	2	35	0	35	5.00	[0.25; 100.48]	0.1%
**Overall Effect**		**1603**		**1603**	**1.12**	**[0.69; 1.83]**	**14.6%**
Heterogeneity: $I^2 = 58\%$, $\tau^2 = 0.2450$, $p < 0.01$							
**Overall Effect**		**15987**		**15987**	**1.01**	**[0.92; 1.12]**	**100.0%**
Heterogeneity: $I^2 = 75\%$, $\tau^2 = 0.0258$, $p < 0.01$							
Test for subgroup differences: $\chi^2_1 = 0.18$, df = 1 ($p = 0.67$)							

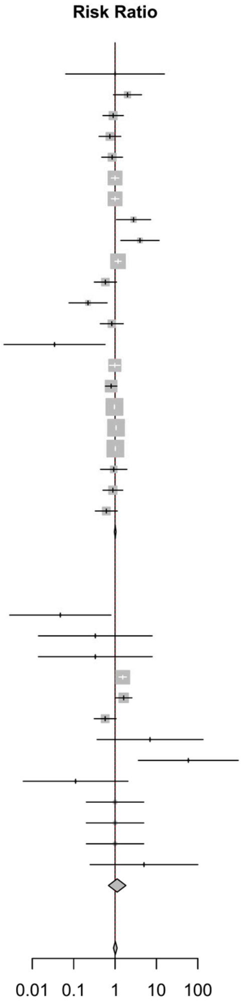

Figure S16. The forest plot represents the subgroup meta-analysis by study quality, with RBPT as reference (control), in sheep. [6,8,9,41,52,56,67,69,74,77,85,89-91,100,101,108,112,114,116,117,128]

Study		Experimental Events	Experimental Total	Control Events	Control Total	RR	95%-CI	Weight
**TestGroup = Agglutination−Rapid**								
Waringa et al.,(2023)	FBAT	9	52	3	52	3.00	[0.86; 10.46]	2.1%
Waringa et al.,(2023)	FBAT	30	84	13	84	2.31	[1.30; 4.11]	2.9%
Waringa et al.,(2023)	FBAT	5	30	1	30	5.00	[0.62; 40.28]	1.3%
**Overall Effect**			**166**		**166**	**2.52**	**[1.52; 4.19]**	**6.3%**
Heterogeneity: $I^2 = 0\%$, $\tau^2 = 0$, $p = 0.75$								
**TestGroup = Culture**								
Almashhadany et al., 2022	Culture	31	325	40	325	0.78	[0.50; 1.21]	3.0%
Yang et al. 2019	Culture	5	154	92	154	0.05	[0.02; 0.13]	2.5%
**Overall Effect**			**479**		**479**	**0.21**	**[0.02; 2.85]**	**5.5%**
Heterogeneity: $I^2 = 96\%$, $\tau^2 = 3.4065$, $p < 0.01$								
**TestGroup = CFT**								
Getahun et al., 2022	CFT	2	166	7	166	0.29	[0.06; 1.36]	1.8%
**TestGroup = Agglutination−Slow**								
Obaidat et al. 2022	BRUCELLACAPT	9	938	56	938	0.16	[0.08; 0.32]	2.7%
Islam et al., 2022	SPAT	3	53	2	53	1.50	[0.26; 8.62]	1.6%
Govindasamy et al. 2021	BRUCELLACAPT	15	230	23	230	0.65	[0.35; 1.22]	2.8%
Yang et al. 2019	SAT	126	154	92	154	1.37	[1.18; 1.59]	3.1%
Proch et al. 2018	SAT	67	279	61	279	1.10	[0.81; 1.49]	3.1%
Sharma et al.(2016)	SAT	11	121	12	121	0.92	[0.42; 2.00]	2.7%
Germeraad et al.(2016)	MAT	0	599	1	599	0.33	[0.01; 8.17]	0.7%
Mangalgi et al.(2015)	SAT	127	1733	182	1733	0.70	[0.56; 0.87]	3.1%
Mangalgi et al.(2015)	2−ME	102	1733	182	1733	0.56	[0.44; 0.71]	3.1%
YÜKSEKKAYA et al. 2014	SAT	34	1000	0	1000	69.00	[4.24; 1123.89]	0.9%
YÜKSEKKAYA et al. 2014	MPAT	22	1000	0	1000	45.00	[2.73; 740.80]	0.9%
Priyadarshini et al. 2013	SAT	3	88	5	88	0.60	[0.15; 2.43]	1.9%
Din et al. 2013	SAT	9	150	14	150	0.64	[0.29; 1.44]	2.6%
Din et al. 2013	SPAT	11	150	14	150	0.79	[0.37; 1.67]	2.7%
**Overall Effect**			**8228**		**8228**	**0.82**	**[0.55; 1.23]**	**32.0%**
Heterogeneity: $I^2 = 86\%$, $\tau^2 = 0.3779$, $p < 0.01$								
**TestGroup = PCR**								
Yousaf et al., 2021	PCR	33	218	37	218	0.89	[0.58; 1.37]	3.0%
Mahmood et al.(2016)	PCR	42	110	5	110	8.40	[3.45; 20.43]	2.5%
**Overall Effect**			**328**		**328**	**2.64**	**[0.29; 23.77]**	**5.5%**
Heterogeneity: $I^2 = 95\%$, $\tau^2 = 2.3878$, $p < 0.01$								
**TestGroup = Primary−Binding−Assay**								
Cadmus et al., 2021	c−ELISA	0	137	1	137	0.33	[0.01; 8.11]	0.7%
Govindasamy et al. 2021	I−ELISA	48	230	23	230	2.09	[1.31; 3.31]	3.0%
Ghugey et al., 2021	I−ELISA	7	382	5	382	1.40	[0.45; 4.37]	2.2%
Yusuf et al., 2021	c−ELISA	3	307	2	307	1.50	[0.25; 8.91]	1.6%
Lukambagire et al., 2021	c−ELISA	35	218	19	218	1.84	[1.09; 3.12]	2.9%
Holt et al., 2021	ELISA	56	585	17	585	3.29	[1.94; 5.60]	2.9%
Munyua et al., 2021	I−ELISA	5	236	4	236	1.25	[0.34; 4.60]	2.1%
Munyua et al., 2021	I−ELISA	39	236	4	236	9.75	[3.54; 26.85]	2.4%
Mangtany et al., 2020	I−ELISA	30	1801	18	1801	1.67	[0.93; 2.98]	2.9%
Yang et al. 2019	ELISA	147	154	92	154	1.60	[1.40; 1.83]	3.1%
Madut et al.(2018)	c−ELISA	29	87	35	87	0.83	[0.56; 1.23]	3.0%
Awah−Ndukum et al.(2018)	I−ELISA	15	816	8	816	1.88	[0.80; 4.40]	2.6%
Proch et al. 2018	ELISA	55	279	61	279	0.90	[0.65; 1.25]	3.1%
Proch et al. 2018	ELISA	150	279	61	279	2.46	[1.92; 3.15]	3.1%
Sharma et al.(2016)	I−ELISA	20	121	12	121	1.67	[0.85; 3.26]	2.8%
Germeraad et al.(2016)	ELISA	0	599	1	599	0.33	[0.01; 8.17]	0.7%
Shirima et al.(2016)	ELISA	0	82	0	82			0.0%
Mahmood et al.(2016)	c−ELISA	10	110	5	110	2.00	[0.71; 5.66]	2.4%
Mahmood et al.(2016)	FPA	34	110	5	110	6.80	[2.76; 16.74]	2.5%
Dean et al. 2013	ELISA	5	683	3	683	1.67	[0.40; 6.95]	1.9%
Aworh et al. 2013	ELISA	40	224	38	224	1.05	[0.70; 1.58]	3.0%
**Overall Effect**			**7676**		**7676**	**1.79**	**[1.36; 2.35]**	**48.8%**
Heterogeneity: $I^2 = 73\%$, $\tau^2 = 0.2294$, $p < 0.01$								
**Overall Effect**			**17043**		**17043**	**1.30**	**[0.95; 1.78]**	**100.0%**
Heterogeneity: $I^2 = 87\%$, $\tau^2 = 0.8159$, $p < 0.01$								
Test for subgroup differences: $\chi^2_5 = 20.74$, df = 5 ($p < 0.01$)								

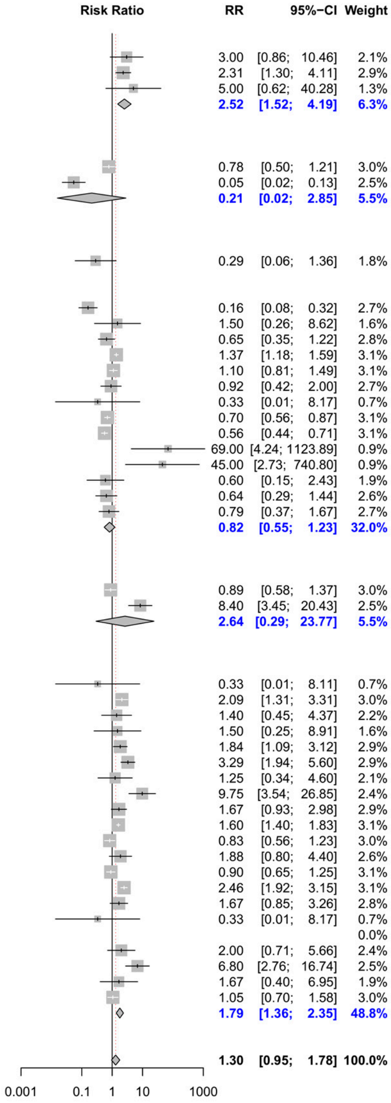

Figure S17. The forest plot depicts the sensitivity analysis based on high-quality studies, with RBPT as reference (control), in humans.

[43,45,46,47,48,49,52,53,54,56,58,59,60,61,62,63,64,65,66,67,68,69,70,71,72,73,183,212]

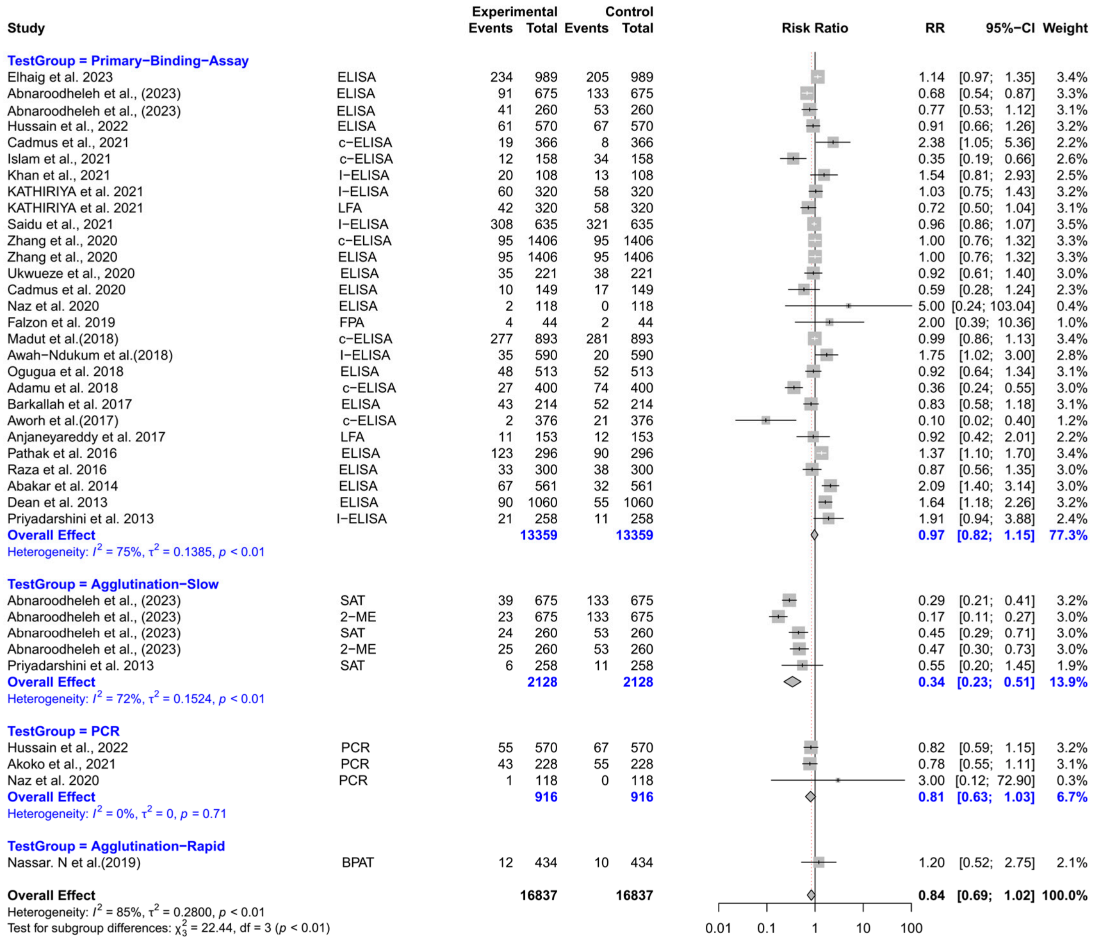

Figure S18. The forest plot depicts the sensitivity analysis based on high-quality studies, with RBPT as reference (control), in cattle. [58,62,69,70,72,87,88,89,90,91,92,93,94,96,97,98,100,102,103,105,106,107,112,115]

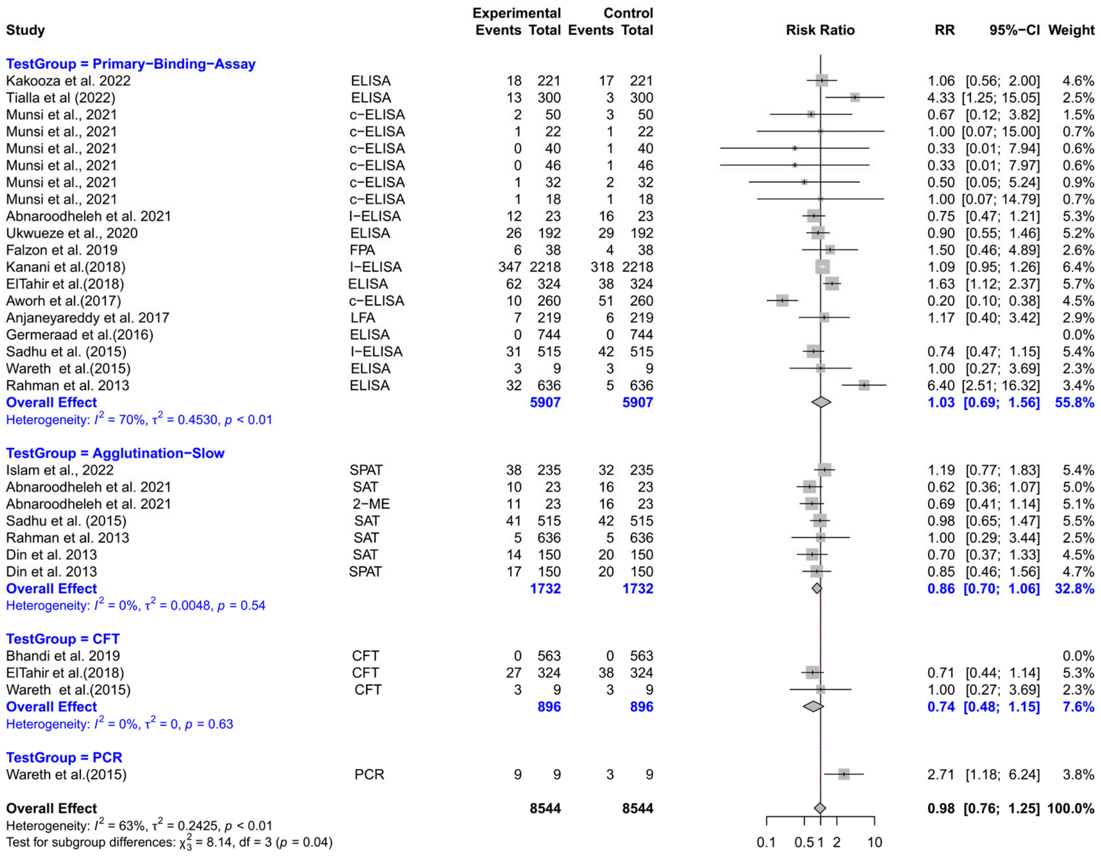

Figure S19. The forest plot depicts the sensitivity analysis based on high-quality studies, with RBPT as reference (control), in goats.

[49,54,59,96,100,105,106,110,117,118,119,120,122,123,126,127,128]

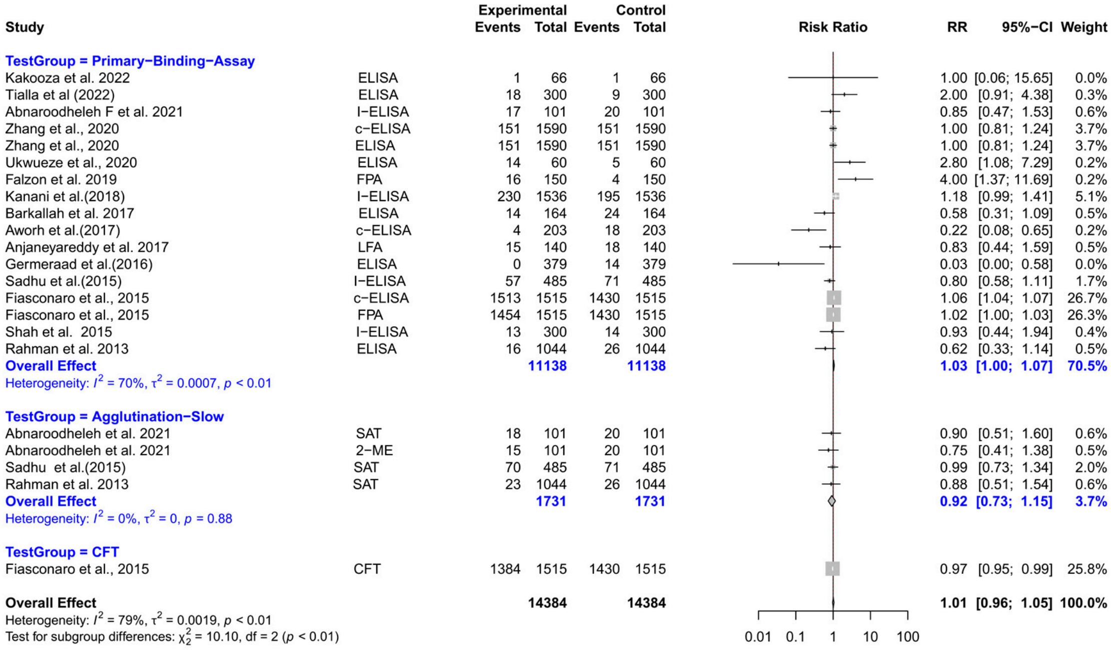

Figure S20. The forest plot depicts the sensitivity analysis based on high-quality studies, with RBPT as reference (control), in sheep. [94,96,100,104,105,106,117,118,120,122,124,126,127,131]
